# Folate status shows no relationship with vitamin B12 but reiterates the urgency for folate fortification in the UK

**DOI:** 10.1007/s00394-025-03796-6

**Published:** 2025-09-06

**Authors:** Albert Koulman, Timothy Woolley, Kerry S. Jones

**Affiliations:** 1 Nutritional Biomarker Laboratory, MRC Epidemiology Unit, University of Cambridge, Cambridge, UK; 2 Inuvi Diagnostics, Gloucester, UK

**Keywords:** Folate (Vitamin B9), Cobalamin (B12), Holotranscobalamin (HoloTC), Folate deficiency

## Abstract

**Background:**

The UK has a high and increasing prevalence of folate deficiency. The decision to start mandatory folic acid fortification has not yet been implemented. Concern has been raised about the effect of high folate on vitamin B12 status.

**Methods:**

The prevalence of folate deficiency was assessed based on the serum folate concentrations from 47,240 samples collected between August 2023 and January 2025 (provided to us as anonymised data, measured in a UK-based contract laboratory using an immunochemical analyser). In 39,374 individuals, both serum folate and holotranscobalamin (holoTC) (‘active’ vitamin B12) concentrations were available, and were used to determine if high folate status had a negative impact on active vitamin B12 concentrations.

**Results:**

For women of reproductive age (16–50 y), 72.7% had serum folate concentration lower than 24.3 nM/L for protection against neural tube defects. For young adult women (21–25 y) this percentage was 85.5%. The top decile, across all samples (mean serum folate = 43.9 nM/L, range 38.4– > 45 nM/L, n = 3935), had on average also a high holoTC concentration (119.2 pM/L) and no increased risk of vitamin B12 deficiency.

**Conclusion:**

Young women are especially a risk of low folate status. We found no evidence that high folate affects vitamin B12 status.

**Supplementary Information:**

The online version contains supplementary material available at 10.1007/s00394-025-03796-6.

## Introduction

Deficiency in folate (vitamin B9) and cobalamin (vitamin B12) can cause megaloblastic anemia. Low folate status also exposes the developing fetus to the risk for developing neural tube defects. Recently we reported a high and rising prevalence of folate deficiency in the UK population [1]. This work was based on the nationally-representative data collected as part of the UK National Diet and Nutrition Survey (NDNS), which until 2023, was limited to a sample size of approximately 500 participants per year for the measurement of folate status.

The poor folate status of the UK population and especially of women of reproductive age [2] has resulted in the decision to introduce mandatory folic acid fortification of non-wholemeal wheat flour, which will be implemented by the end of 2026 [3].

Lack of folate or vitamin B12 can cause megaloblastic anemia, but the condition caused by vitamin B12 deficiency can be recovered by folic acid supplementation. Consequently, there has been concern that high folic acid intake (achieved through supplementation) can mask underlying vitamin B12 deficiency with later neurological outcomes, although the evidence to date suggests this is unlikely with folic acid fortification [4].

It has also been hypothesized that folic acid may directly impact the metabolism of holotranscobalamin (holoTC), the active form of vitamin B12. [5, 6]. We therefore used recent and large-scale data, measured in a UK-based contract laboratory, providing serum folate and serum holoTC concentrations to assess folate status and to test if individuals with high serum folate concentrations were at increased risk for vitamin B12 deficiency.

## Method

Serum folate concentrations were measured in 47,240 samples of anonymised individuals.. The samples were collected from August 2023 to Jaunuary 2025. Only age (in 5-year brackets) and sex were available. Samples were collected from participants who had self-referred for testing, but there was no data for the reason, nutritional status nor other health outcomes or measurements. It is possible that some individuals could have provided more than one sample across these 18 months of data collection.

All the samples were measured in a UK-based contract laboratory using an immunochemical analyser (Roche Elecsys, Roche, UK). For 39,374 of the individuals for which serum folate was measured, serum holoTC concentration was also available. Serum folate concentrations that were above the limit of quantitation (> 45.3 nM/L), were included in the calculations as 45.3 nM/L. Similarly, holoTC concentration above the limit of quantitation (> 150 pM/L) were included in the calculation as 150 pM/L).

The following thresholds and cut-offs were used for to classify individuals: < 13 nM/L indicates a low folate status and increased risk of megaloblastic anemia, < 7 nM/L were folate deficient. For full protection against neural tube defects women of reproductive age need to have a serum folate of 24.3 nM/L [2]. The cut-off for vitamin B12 deficiency was a holoTC concentration of < 35 pM/L. Very high folate concentration, > 40 nM/L, was used to indicate regular and/or high dose folic acid supplementation.

We used descriptive analysis to show the occurrence of folate deficiency by age and sex groups. We also calculate mean with standard deviation and median with interquartile range for folate and holoTC concentrations in the age groups by sex. Formal statistical analysis was not performed due to the convenience nature of this sample set.

## Results

The results of 47,240 serum folate concentrations showed that almost 1 in 8 individuals were deficient (< 7 nM/L) for folate (across all ages and sex). Within this dataset there were 17,868 samples from women of reproductive age (16–50 y), for which 72.7% had a serum folate concentration lower than 24.3 nM/L. For young adult women (21–25 y) this percentage was 85.5%. We saw that 10.1% of women between 25 and 40 y reach a high folate status (> 40 nM/L), while for men this was 4.5% (Table 1).

**Table 1:** Prevalence of folate concentration less than thresholds and cut-offs by age in UK women of reproductive age (data collected between August 2023 to January 2025)

Age, y	Sex	Total (n)	Number below serum folate thresholds^a^			Percentage below serum folate thresholds		
			< 24.3 nM/L	< 13 nM/L	< 7 nM/L	< 24.3 nM/L	< 13 nM/L	< 7 nM/L
16–90	m,f	47,240	34,731	19,054	5709	73.52%	40.33%	12.09%
16–90	m	21,352	16,494	8964	2540	77.25%	41.98%	11.90%
16–90	f	25,891	18,239	10,091	3169	70.45%	38.97%	12.24%
16–50	f	17,868	12,997	7513	2532	72.74%	42.05%	14.17%
16–20	f	331	270	180	82	81.57%	54.38%	24.77%
21–25	f	1033	883	613	272	85.48%	59.34%	26.33%
26–30	f	2322	1875	1185	433	80.75%	51.03%	18.65%
31–35	f	3479	2537	1471	514	72.92%	42.28%	14.77%
36–40	f	3891	2664	1478	473	68.47%	37.99%	12.16%
41–45	f	3781	2626	1441	432	69.45%	38.11%	11.43%
46–50	f	3031	2142	1145	326	70.67%	37.78%	10.76%

^a^Thresholds: < 24.3 nM/L is the threshold for increased risk of neural tube defect, < 13 nM/L is the threshold for increased risk of megaloblastic anemia, < 7 nM/L is the cut-off for folate deficiency

For all individuals (n = 47,240), 42% had serum folate concentration below 13 nM/L, putting them at increased risk for megaloblastic anemia. These percentages were higher in younger adults (both male and female).

In the population studied, women aged 21 to 25 years had the poorest folate status, with over a quarter (26.3%) having a serum folate level below 7 nM/L. In comparison, 22.2% of males in the same age group had folate serum levels below 7 nM/L. For women that are planning to become pregnant it is advised to take folic acid supplements. Within this age groups we only saw a small percentage (3.7%) with very high folate concentrations (> 40 nM/L), which was slightly higher than in males (2.8%) of the same age groups (See Suppl Table 1).

For 39,374 individuals, both serum folate and holoTC concentrations were measured (see Suppl Table 2). The prevalence of the vitamin B12 deficiency (serum holoTC < 35 pM/L, [7, 8]) in this population was ~ 1.4% (n = 553). Among these individuals with the highest serum folate concentration (≥ 45.4 nM/L (n = 2385) there were only 9 (0.4%) individuals with low holoTC serum concentration (< 35 pM/L), while among all those with a healthy folate concentration (> 13 nM/L, n = 28,813) there were 175 individuals with low holoTC (0.7%).

## Discussion

Folate deficiency is common in the UK, with more than one in five young adults having serum folate concentration < 7 nM/L. Most women of reproductive age have a folate status during pregnancy that is insufficient to protect against neural tube defects.

These results highlight the importance of implementing mandatory folic acid fortification as planned by the UK government in 2026. Our data show that within this population only 9% of the individuals have folate concentrations above 40 nM/L and would therefore receive limited benefit from the fortification. In contrast, more than 40% of adults (with folate concentration < 13 nM/L) may benefit from an increased folate status. This percentage remains similar in the age group where voluntary supplementation is recommended, such as women aged 16–50 years who may become pregnant. Only in elderly people (> 60 years) are there more people with a high folate status, which appears to increase with age.

Mandatory fortification is therefore unlikely to result in excessively high folate concentrations in most people, even among those already taking supplements. This highlights the importance to keep the advice for women who want to become pregnant to use folic acid supplementation. Furthermore, from our results there is no evidence of disturbed vitamin B12 metabolism in people with high serum folate.

This study is not nationally representative, and the participants were mainly self-referring and fee-paying (personal communication of Inuvi Diagnostics). The overall picture of folate status was very comparable to recent results of the NDNS [1, 9]. It is possible that there are some biases in the data. Individuals referred themselves for this analysis and might have considered themselves at risk, which could have inflated the number of individuals with a low status. This could explain the high prevalence of folate deficiency, although we do not observe the same for vitamin B12 status. Another possible bias—which could have reduced the numbers of participants with a low folate status- is that individuals that self-refer are more likely to have a higher disposable income and higher social economic status. Several studies have shown that both folate and vitamin B12 status are correlated with socioeconomic status and income [1, 9, 10]. Furthermore, only age bracket and sex was available for the participants. However, this is a recent and large data set from the UK and provides valuable data contributing to understanding of folate and vitamin B12 status. The study is also limited to serum folate and holoTC. Measurements of red blood cell folate, homocysteine and methylmalonic acid (MMA) could enable a better diagnosis of folate and B12 status, however these measurements were not available for these participants.

It remains unclear why so many young people are folate deficient. Within the NDNS, there are insufficient data to assess folate status with the age group granularity possible in this dataset [1]. However, NDNS data from 11 to 18 year olds does indicate lower folate intake in this age group compared with older age groups and folate intake is lower in females compared to males [11]. More research is needed on adolescents and young adults to understand how diet and lifestyle affect essential nutrients like folate.

Overall, these results support the need to introduce folic acid fortification to support population folate status, particularly for women of reproductive age, with benefits including the prevention of neural tube defects [12, 13] and a reduction in other health risks.

## Supplementary Information

Below is the link to the electronic supplementary material.

Supplementary Material 1
