# Supplementary material for: Folate status shows no relationship with vitamin B12 but reiterates the urgency for folate fortification in the UK
Source: Eur J Nutr. 2025 Sep 6;64(6):272. doi: 10.1007/s00394-025-03796-6 (PMC12414022; doi:10.1007/s00394-025-03796-6)
Supplement: Supplementary file 1 — Supplementary Material 1 [file 394_2025_3796_MOESM1_ESM.docx]

Folate status shows no relationship with vitamin B12 but reiterates the urgency for folate fortification in the UK.

Albert Koulman^1*^, Timothy Woolley^2^, Kerry S. Jones^3^

^1^: Nutritional Biomarker Laboratory, MRC Epidemiology Unit, University of Cambridge, Cambridge, UK

^2^: Inuvi Diagnostics, Gloucester, UK

Supplementary information

Suppl. Table 1. Prevalence of folate concentration less than threholds and cut-offs by age in UK (data collected between August 2023 to January 2025).

|  |  |  | Number below serum folate thresholds^1^ | | | Percentage below serum folate thresholds | | | High folate^2^ | |
| --- | --- | --- | --- | --- | --- | --- | --- | --- | --- | --- |
| Age (y) | Sex | Total (n) | <24.3 nM/L | <13 nM/L | <7 nM/L | <24.3 nM/L | <13 nM/L | <7 nM/L | >40 nM/L (n ,%) | |
| 16-90 | m,f | 47240 | 34731 | 19054 | 5709 | 73.5% | 40.3% | 12.1% | 4226 | 8.9% |
| 16-90 | f | 25891 | 18239 | 10091 | 3169 | 70.4% | 39.0% | 12.2% | 2872 | 11.1% |
| 16-50 | f | 17868 | 12997 | 7513 | 2532 | 72.7% | 42.0% | 14.2% | 1783 | 10.0% |
| 16-20 | f | 331 | 270 | 180 | 82 | 81.6% | 54.4% | 24.8% | 20 | 6.0% |
| 21-25 | f | 1033 | 883 | 613 | 272 | 85.5% | 59.3% | 26.3% | 38 | 3.7% |
| 26-30 | f | 2322 | 1875 | 1185 | 433 | 80.7% | 51.0% | 18.6% | 141 | 6.1% |
| 31-35 | f | 3479 | 2537 | 1471 | 514 | 72.9% | 42.3% | 14.8% | 372 | 10.7% |
| 36-40 | f | 3891 | 2664 | 1478 | 473 | 68.5% | 38.0% | 12.2% | 468 | 12.0% |
| 41-45 | f | 3781 | 2626 | 1441 | 432 | 69.5% | 38.1% | 11.4% | 440 | 11.6% |
| 46-50 | f | 3031 | 2142 | 1145 | 326 | 70.7% | 37.8% | 10.8% | 307 | 10.1% |
| 51-55 | f | 2711 | 1887 | 953 | 256 | 69.6% | 35.2% | 9.4% | 306 | 11.3% |
| 56-60 | f | 2108 | 1402 | 705 | 191 | 66.5% | 33.4% | 9.1% | 266 | 12.6% |
| 61-65 | f | 1354 | 830 | 402 | 81 | 61.3% | 29.7% | 6.0% | 201 | 14.8% |
| 65-70 | f | 944 | 567 | 249 | 58 | 60.1% | 26.4% | 6.1% | 165 | 17.5% |
| 71-75 | f | 506 | 315 | 145 | 22 | 62.3% | 28.7% | 4.3% | 83 | 16.4% |
| 76-80 | f | 273 | 170 | 89 | 23 | 62.3% | 32.6% | 8.4% | 44 | 16.1% |
| 81-85 | f | 86 | 49 | 22 | 5 | 57.0% | 25.6% | 5.8% | 18 | 20.9% |
| >85 | f | 36 | 18 | 11 | 1 | 50.0% | 30.6% | 2.8% | 5 | 13.9% |
| 16-90 | m | 21352 | 16494 | 8964 | 2540 | 77.2% | 42.0% | 11.9% | 1354 | 6.3% |
| 16-20 | m | 271 | 231 | 163 | 56 | 85.2% | 60.1% | 20.7% | 13 | 4.8% |
| 21-25 | m | 1255 | 1092 | 732 | 279 | 87.0% | 58.3% | 22.2% | 35 | 2.8% |
| 26-30 | m | 2616 | 2237 | 1363 | 489 | 85.5% | 52.1% | 18.7% | 87 | 3.3% |
| 31-35 | m | 3733 | 3040 | 1719 | 481 | 81.4% | 46.0% | 12.9% | 162 | 4.3% |
| 35-40 | m | 3581 | 2741 | 1467 | 396 | 76.5% | 41.0% | 11.1% | 204 | 5.7% |
| 41-45 | m | 2961 | 2203 | 1138 | 292 | 74.4% | 38.4% | 9.9% | 214 | 7.2% |
| 46-50 | m | 2038 | 1465 | 701 | 164 | 71.9% | 34.4% | 8.0% | 182 | 8.9% |
| 51-55 | m | 1718 | 1259 | 622 | 143 | 73.3% | 36.2% | 8.3% | 129 | 7.5% |
| 56-60 | m | 1275 | 922 | 437 | 91 | 72.3% | 34.3% | 7.1% | 97 | 7.6% |
| 61-65 | m | 827 | 566 | 264 | 58 | 68.4% | 31.9% | 7.0% | 107 | 12.9% |
| 66-70 | m | 532 | 373 | 184 | 43 | 70.1% | 34.6% | 8.1% | 56 | 10.5% |
| 71-75 | m | 294 | 205 | 95 | 29 | 69.7% | 32.3% | 9.9% | 33 | 11.2% |
| 76-80 | m | 166 | 115 | 61 | 10 | 69.3% | 36.7% | 6.0% | 19 | 11.4% |
| 81-85 | m | 51 | 32 | 12 | 6 | 62.7% | 23.5% | 11.8% | 6 | 11.8% |
| >85 | m | 31 | 11 | 5 | 2 | 35.5% | 16.1% | 6.5% | 10 | 32.3% |

^1^: Thresholds: <24.3 nM/L is threshold for neural tube defect risk, <13 nM/L is threshold for megaloblastic anemia risk, <7 nM/L is threshold for folate deficiency.

^2^: Threshold: <40 nM/L is suggesting the use of dietary supplements containing folic acid.

Suppl table 2, Median and mean concentration of serum folate and active B12(HoloTC) in participants with both measurements by age group and sex.

| Age (y) | Sex | Total (n) | Folate (nm/l) | | Active b12 (holotc) (pm/l) | |
| --- | --- | --- | --- | --- | --- | --- |
|  |  |  | Median (IQR) | Mean (SD) | Median (IQR) | Mean (SD) |
| 16-20 | f | 260 | 12 (7,20) | 15 (11) | 78 (56,119) | 87 (38) |
| 21-25 | f | 802 | 12 (7,18) | 15 (10) | 75 (55,111) | 84 (38) |
| 26-30 | f | 1,964 | 13 (8,21) | 16 (11) | 77 (57,116) | 87 (37) |
| 31-35 | f | 2,999 | 16 (9,26) | 19 (12) | 80 (58,121) | 89 (38) |
| 36-40 | f | 3,342 | 17 (10,28) | 20 (13) | 83 (61,124) | 91 (37) |
| 41-45 | f | 3,107 | 17 (10,28) | 20 (12) | 89 (63,134) | 95 (38) |
| 46-50 | f | 2,259 | 17 (11,28) | 20 (12) | 92 (65,138) | 98 (38) |
| 51-55 | f | 1,899 | 19 (11,29) | 21 (12) | 97 (68,146) | 101 (38) |
| 56-60 | f | 1,529 | 19 (11,30) | 21 (12) | 102 (72,150) | 104 (37) |
| 61-65 | f | 1,081 | 19 (12,32) | 23 (12) | 107 (78,150) | 108 (35) |
| 66-70 | f | 758 | 20 (12,34) | 23 (13) | 111 (78,150) | 110 (36) |
| 71-75 | f | 407 | 20 (13,32) | 23 (13) | 108 (74,150) | 107 (37) |
| 76-80 | f | 222 | 18 (11,31) | 22 (13) | 113 (75,150) | 109 (37) |
| 81-85 | f | 67 | 18 (11,39) | 24 (14) | 117 (83,150) | 113 (36) |
| >86 | f | 26 | 21 (13,36) | 24 (13) | 106 (92,150) | 110 (33) |
| 16-20 | m | 242 | 11 (8,17) | 14 (10) | 89 (65,122) | 94 (35) |
| 21-25 | m | 1,129 | 12 (8,18) | 14 (9) | 101 (72,150) | 104 (37) |
| 26-30 | m | 2,352 | 13 (8,20) | 15 (9) | 97 (68,144) | 101 (38) |
| 31-35 | m | 3,326 | 14 (9,21) | 16 (10) | 97 (69,142) | 101 (37) |
| 36-40 | m | 3,164 | 15 (10,23) | 18 (11) | 95 (70,141) | 100 (36) |
| 41-45 | m | 2,567 | 16 (10,25) | 19 (11) | 96 (70,140) | 101 (36) |
| 46-50 | m | 1,759 | 17 (11,26) | 20 (11) | 93 (68,134) | 99 (36) |
| 51-55 | m | 1,488 | 16 (11,25) | 19 (11) | 89 (65,129) | 95 (36) |
| 56-60 | m | 1,057 | 17 (11,26) | 19 (11) | 85 (64,123) | 93 (36) |
| 61-65 | m | 686 | 18 (11,28) | 21 (12) | 89 (66,129) | 96 (36) |
| 66-70 | m | 447 | 17 (11,28) | 20 (12) | 91 (65,127) | 96 (36) |
| 71-75 | m | 235 | 18 (11,27) | 20 (12) | 95 (69,138) | 99 (37) |
| 76-80 | m | 133 | 17 (11,28) | 21 (12) | 87 (66,120) | 94 (34) |
| 81-85 | m | 41 | 21 (13,31) | 23 (13) | 130 (74,150) | 111 (42) |
| >86 | m | 26 | 34 (20,45) | 31 (13) | 115 (75,150) | 109 (43) |
